# Prone positioning effect on tracheal intubation rate, mortality and oxygenation parameters in awake non-intubated severe COVID-19-induced respiratory failure: a review of reviews

**DOI:** 10.1186/s40001-024-01661-6

**Published:** 2024-01-20

**Authors:** Sepideh Tahsini Tekantapeh, Nader D. Nader, Morteza Ghojazadeh, Fatemeh Fereidouni, Hassan Soleimanpour

**Affiliations:** 1 Student Research Committee, Department of Rheumatology, Tabriz University of Medical Sciences, Tabriz, Iran; 2 Departments of Anesthesiology and Surgery, Jacobs School of Medicine and Biomedical Sciences, University at Buffalo, Buffalo, NY USA; 3 Research Center for Evidence-Based Medicine, Tabriz University of Medical Sciences, Tabriz, Iran; 4 Student Research Committee, Tabriz University of Medical Sciences, Tabriz, Iran; 5 Integrated Aging Research Center, Tabriz University of Medical Sciences, Tabriz, Iran

**Keywords:** Awake, Acute hypoxic respiratory failure, COVID-19, Prone position, Supine position

## Abstract

**Background:**

Prone positioning (PP) is a low-cost method with minimal risk to the patient that improves the oxygenation of patients with acute hypoxic respiratory failure (AHRF) due to COVID-19 pneumonia, thereby reducing their need for tracheal intubation (TI) and transferring to the intensive care unit (ICU). We aimed to overview the results of all previous systematic reviews and meta-analyses to examine the net effect of PP on oxygenation, the rate of TI and mortality in COVID-19 patients.

**Methods:**

We searched PubMed, Scopus, Web of Science, Google Scholar, and Cochrane Library databases from December 2019 through 2022 without publication language restriction for systematic reviews and meta-analysis studies on PP vs. supine position (SP) in conscious patients with hypoxic respiratory failure COVID-19. After study selection, data were extracted from published meta-analyses and pooled by comprehensive meta-analysis (CMA) software version 2.2.064 to achieve effect sizes. They were analyzed for TI and mortality rates dichotomous variables, and the results were shown as pooled odds ratios (OR) with a 95% confidence interval (CI). Continuous variables such as oxygenation indices (PaO2/FiO2 and SpO2) were also analyzed, and the data were shown as mean differences (MD) with lower and upper CI. The level of statistical significance was set at *p* ≤ 0.05.

**Results:**

Twelve systematic reviews and meta-analyses with 19,651 patients and six systematic reviews with 2,911 patients were included in this Review of Reviews (total: 22,562). PP treatment significantly reduced the rate of TI (OR = 0.639, %95 CI (0.492, 0.829); *P*-value = 0.001) and decreased mortality (OR = 0.363, %95 CI (0.240, 0.549), *P*-value < 0.001). There was no difference in PaO2/FiO2 (MD = 3.591[− 40.881, 48.062]; *P*-value = 0.874) and SpO2 percent (MD = 1.641[− 4.441, 7.723]; *P*-value = 0.597).

**Conclusion:**

Prone positioning can be recommended in conscious ICU patients with COVID-19 pneumonia to reduce mortality and intubation.

*Systematic review registration:* PROSPERO registration number: CRD42022326951. Registered 25 April 2022.

**Graphical Abstract:**

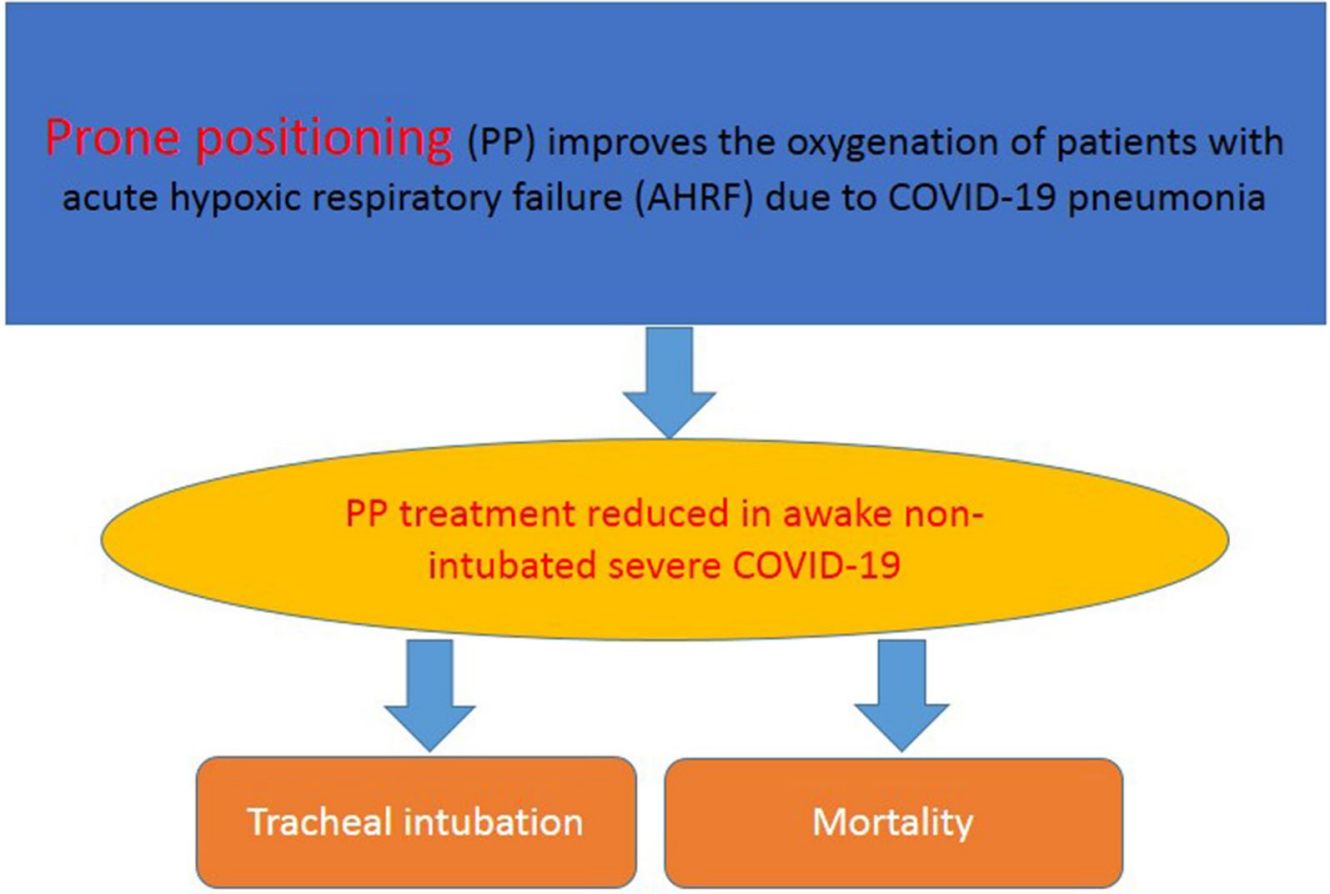

**Supplementary Information:**

The online version contains supplementary material available at 10.1186/s40001-024-01661-6.

## Introduction

Due to the rapid spread of severe acute respiratory syndrome coronavirus 2 (SARS-CoV-2) in the severe COVID-19 pneumonia during the pandemic, significant alveolar involvement progresses to acute respiratory distress syndrome (ARDS) [1, 2]. With the subsequent reduction in lung capacity and the development of severe ventilation-perfusion (*V*/*Q*) mismatch, the induced lung injury leads to severe shunting and oxygen desaturation [3]. Prone positioning (PP) is among the physical methods which have effectively improved pulmonary ventilation and the oxygenation profile [4–6]. The prone position is a low-cost, low-risk method for reducing the need for tracheal intubation and transfer to the Intensive Care Unit (ICU) in awake non-intubated patients with severe hypoxic COVID-19 [7–11].

An increase in lung volumes occurs due to PP, especially in the lower lobes [12, 13]. Several observational studies have been published about the positive effects of PP in conscious non-intubated patients with severe COVID-19 pneumonia and oxygen-resistant hypoxemia [14–16]. Several clinical trials have confirmed that PP improves oxygenation and reduces the respiratory rate (RR) in awake patients [7, 11]. In patients with ARDS due to severe COVID-19 infection with the involvement of the lungs, one way to control the extent of pulmonary shunting and correct the resulting V/Q mismatch is to use PP by placing them in a face-down position on their abdomen. There is evidence that the interstitial fluid congestion caused by the accumulation of perialveolar fluid and pulmonary airway pressures are reduced in PP, thereby improving oxygenation and decreasing the need for tracheal intubation (TI) and mechanical ventilation in awake patients [17].

The range for the duration of PP varies from 30 min to 2 h in each position [18]. Prasad et al. have recommended periods as long as 2–3 h up to 4–5 h per day [19]. Following the application of PP, the clinical condition of the patients recovers rapidly as the basilary atelectasis ceases to progress in the lungs [20]. In a case–control study of 600 conscious, non-intubated COVID-19 patients hospitalized in three principal urban hospitals, there was no increase in the rate of TI with mechanical ventilation and mortality in those treated with PP [21].

This Review of Reviews aims to combine the existing meta-analyses and systematic reviews and examines the effect of PP on the rates of TI and mortality along with the oxygenation profile in a large number of awake Covid-19 patients with increased statistical power. Several meta-analyses have systematically reviewed PP in awake patients with acute hypoxemic respiratory failure caused by moderate to severe COVID-19 against the traditional supine position (SP) with varying degrees of head elevation.

## Methods

Our Review of Reviews were performed according to the Preferred Reporting Items for Systematic Reviews and Meta-Analyses (PRISMA) checklist. The protocol was prospectively registered at PROSPERO, the International Prospective Registry of Systematic Reviews (ID: CRD42022326951).

### Search strategy

A database search by medical subject heading (MESH) terms and keywords used for each database according to Additional file 1: Table S1 was done comprehensively from December 1st, 2019, to July 1st, 2022, in PubMed, Scopus, Web of Science, the Cochrane Library, and Google Scholar, and some references cited in relevant meta-analysis studies selected manually. After deleting the duplicate articles, the titles and abstracts of the articles were reviewed for APP in hypoxic COVID-19 patients without any language restrictions and type of published articles. These steps were performed by two authors, S.T. and H.S. In the inclusion or exclusion of articles, if the problems did not solve by discussion, to disagreement management, the third author, M.G should have interfered and solved. Finally, eighteen studies were selected, including twelve systematic reviews and meta-analyses and six systematic reviews.

### Inclusion criteria

Systematic review studies or systematic review and meta-analysis studies meeting the following criteria were included in this overview and meta-analysis study:

- Systematic reviews and meta-analyses in which patients over 18 years of age, no gender restriction, and acute hypoxemic respiratory failure because of severe COVID-19 pneumonia were compared for the effect of PP vs. SP on TI rates, mortality, and oxygenation improvement parameters.
- The data have been meta-analyzed.
- There should be no language restrictions on the selection of articles.

Participant Intervention-Comparison-Outcome (PICO) in this study is defined as follows:

Participants (P): awake adult severe COVID-19 patients with acute hypoxic respiratory failure;

Interventions (I): PP; Comparison group(C): SP or standard of care (SOC); Outcome measures (O): TI rate (dichotomous), the mortality rate (dichotomous) and oxygenation parameters such as the ratio of arterial partial pressure of oxygen and fraction inspiratory oxygen (PaO2/FiO2) and pulse-oximetric saturation of oxygen (SpO2) as continuous outcome variables.

To make sure we haven't lost any data, the data related to the effects of PP in non-intubated conscious patients were also extracted from the studies that evaluated the impact of PP vs. SP in non-intubated and intubated patients. Studies that examined PP compared to SP in intubated and unconscious patients or children under 18 years of age were excluded.

### Data extraction

The following items were extracted from systematic reviews and meta-analyses. Types and number of observational or clinical trial studies each included systematic reviews and meta-analysis studies, number of patients in each study, variables and outcomes, methodology, confidence interval (CI), mean difference (MD), and odds ratio (OR), heterogeneity of each study in terms of *I*-squared index (*I*^2^) and *P*-values, assess the quality of studies and the risk of bias (ROB). Two authors performed data extraction individually; if necessary, the third author was consulted. Data were then finalized and with the agreement of all authors.

### Statistical analysis

The relative risk values for tracheal intubation and mortality variables and the mean difference values before and after the intervention (prone positioning) for PaO2/FiO2 and SpO2 variables were selected as effect indicators.

The *I*^2^ was used to determine the heterogeneity among the studies. *I*^2^ values greater than 0.50 were considered heterogeneous. By computing the *I*^2^ for heterogeneity, when there was no statistically significant difference in heterogeneity, a fixed effect model was utilized to analyze the data. In the absence of this, a random effect model was used. A funnel diagram and Begg’s test were utilized to examine diffusion bias. A probability value of less than 0.05 was considered a significant level. All data analyses were conducted with CMA software version 2.2.064 (Biostat Inc., Englewood, NJ, USA).

## Results

### Search results

In the initial search we found 903 articles. After careful stepwise review according to the PRISMA flow diagram, eighteen systematic reviews were included, including twelve meta-analyses and six systematic narrative reviews. Meta-analyses in which PP was examined on intubated patients and under mechanical ventilation were excluded.

**Fig. 1:**
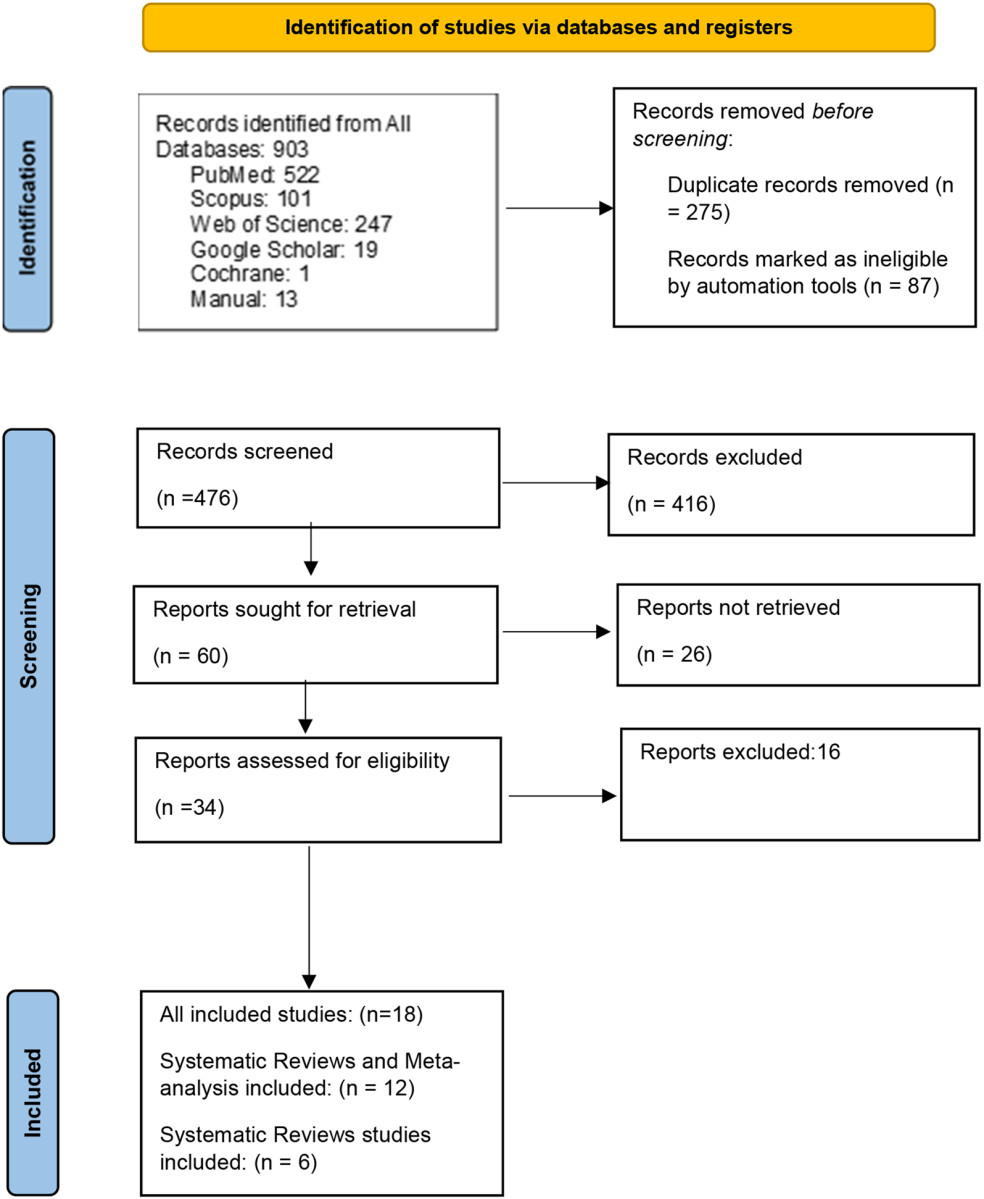
PRISMA 2020 flow diagram

How selection of articles and the initial search in the form of a PRISMA chart is shown in Fig. 1.

### Characteristics of included meta-analyses

Table 1 shows the essential characteristics of each included study, including country of origin, database search, type and number of studies entered in each meta-analysis, including clinical trial or observational studies, case reports or case series. The number of patients has been shown in each of the studies (a total of 22,562 patients in twenty included studies, separately 19,651 and 2911 patients in meta-analyses (twelve studies) and systematic reviews (six studies), respectively) [22–39].

**Table 1:** Characteristics included systematic review and meta-analysis studies

Author/year/reference	Study type	Type of review	Country	Database searched	Type of included studies	Included primary studies (*N*)	Study population (age ≥ 18 y)
*Systematic reviews and meta-analyses*							
Fazzini et al., 2021 [18]	Systematic review and meta-analysis	Non-Cochrane	UK	PubMed/MEDLINE, Cochrane Library, Embase, CINAHL, and BMJ Best Practice until August 2021	8 prospective cohorts (2 multicenter) 4 retrospective cohorts (2 multicenter) 2 RCT (2 multicenter)	14	2352
E.X. Chua et al., 2021 [19]	Systematic review and meta-analysis	Non-Cochrane	Malaysia, UK, Egypt	EMBASE, MEDLINE and CENTRAL until March 2021. ClinicalTrials.gov and WHO International Clinical Trials Registry Plat form for any ongoing or unpublished trials. No restriction in publication language and date	Non-randomized trials(1 Single Center Bicentric study, 1 Single Center Pilot study and 1 Retrospective Observational Study) 32 cohort studies(2 multicenter and 30 single center)	35	1712
Schmid et al., 2022 [20]	Systematic review and meta-analysis	Non-Cochrane	Germany, Spain	COVID19.cochrane.org,MEDLINE (PubMed), Embase, ClinicalTrials.gov, WHO International Clinical Trials Registry Platform & medRxiv (searched on 26October 2021), WHO COVID-19 Global literature, WOS, with no language restriction	1 Randomized, Controlled, Multinational, Open-Label Meta-Trial 1 Multicenter Randomized Clinical Trial	2	1196
Pavlov et al., 2022 [21]	Systematic review and meta-analysis	Non-Cochrane	US	MEDLINE, Embase, PubMed, WOS, Scopus, medRixv, ClinicalTrials.gov and Wanfang from January 1, 2020, to August 15, 2020, with language restrictions to English and Chinese	Prospective systematic review and meta-analysis of observational studies	50	2994
Ponnapa Reddy et al., 2021 [22]	Systematic review and meta-analysis	Non-Cochrane	Australia	PubMed, EMBASE, medRxiv, bioRxiv and the coronavirus diseases 2019 living systematic review from december1,2019,to november9,2020	25 observational studies(2 single arm) case series (single center) 23 cohort studies(22 single center and 1 multicenter)	25	758
Sryma PB et al., 2021 [23]	Systematic review and meta-analysis	Non-Cochrane	India	PubMed and EMBASE databases	Randomized controlled studies(7 prospective trials,3 before-after studies, 6 retrospective series)	16	363
M. T. Awad et al., 2021 [24]	Systematic review and meta-analysis	Non-Cochrane	US	PubMed/MEDLINE, Embase, WHO COVID-19, LitCOVID, and WOS from January 1, 2020, until November 30, 2020, not limited by language, study design, or country of origin	1 Single-center Retrospective Cohort Study 1 single-center prospective cohort study 1 multicenter, adjusted cohort study	3	290
Beran et al., 2021 [25]	Systematic review and meta-analysis	Non-Cochrane	US	PubMed/MEDLINE, EMBASE, Cochrane Central Register of RCTs from inception to August 30, 2021and manual search for additional relevant studies using references of the included articles	5 randomized controlled trials (RCTs) 3 prospective cohort study 6 retrospective cohort study	14	3324
Cardona et al., 2021 [26]	Systematic review and meta-analysis	Non-Cochrane	US	PubMed, Embase, and Scopus databases through August 15, 2020	3 prospective cohort study 2 retrospective cohort study 2 prospective case series 11 retrospective case series	18	364
R. S. Cruz et al., 2021 [27]	Systematic review and meta-analysis	Non-Cochrane	Argentina and Chile	MEDLINE, CENTRAL, WOS and Lilacs on August 20th, 2021	randomized controlled trials (RCTs)	7	1401
Jie Li et al., 2022 [28]	Systematic review and meta-analysis	Non-Cochrane	USA	MEDLINE, Embase, PubMed, WOS, Scopus, MedRxiv, BioRxiv, and ClinicalTrials.gov published in English from Jan 1, 2020, to Nov 8, 2021	10 RCTs (three unpublished from ClinicalTrials.gov), 19 observational studies	29	1985 2669
*Meta-analyses*							
W Tan et al., 2021 [29]	Meta-analysis	Non-Cochrane	China	PubMed, EMBASE and the Cochrane Central Register of Controlled Trials from 1 January 2000 to 1 July 2020, With English language restriction	6 cohort studies and 10 case series	16	243
*Systematic reviews*							
G. T. Chilkoti et al., 2021 [30]	Systematic review	Non-Cochrane	India	PubMed, MEDLINE, Embase, and Google Scholar from December 2019 to May 15, 2020	17 original articles 9 case series 10 case reports	36	1385
S. Anand et al., 2020 [31]	Systematic review	Non-Cochrane	India	PubMed, Google Scholar until July 5, 2020	4 prospective cohort studies, 1 cross-sectional, 6 case series and 2 case reports	13	210
Parashar et al., 2021 [32]	systematic review	Non-Cochrane	India	PubMed, EMBASE, Cochrane Central Register (CENTRAL), WOS, Google Scholar, and one trial registry were searched until September 23, 2020, in English language only	14 prospective or 6 retrospective cohort studies and 1 pilot study (19 single arm and 2 with comparison group) (also 23 registered clinical trials without any RCT)	21	698
Alhazzani et al., 2021 [33]	Systematic review	Non-Cochrane	Multicenter (43 experts from 14 countries)	ClinicalTrials.gov	12 prospective cohorts, 18 retrospective cohorts, and 5 case reports (29 of these studies included COVID-19 patients) ongoing 7 clinical trials	35	414
Ko et al., 2021 [34]	Systematic review	Non-Cochrane	Taiwan	Medicine and Cochrane library	5 case series and 1 Prospective uncontrolled noncomparative study	6	140
Senderovich H et al., 2022 [35]	Systematic review	Non-Cochrane	Canada	PubMed, MedRxiv, and JAMA	2 distinct observational studies, 1 observational cohort studies	3	64

*WHO* World Health Organization, *WOS* Web of Science, *RCT* randomized clinical trials

A summary of included study methods covering inclusion and exclusion criteria, characteristics of patients, mean age, and intervention or control categories is fully shown in Table 2.

**Table 2:** Methods of included systematic reviews and meta-analysis studies

Author/year/reference	Methods		Detail of participants	Mean age (yr.)	Intervention/control
	Inclusion criteria	Exclusion criteria			
*Systematic reviews and meta-analyses*					
Fazzini et al., 2021 [18]	Adult patients (age ≥ 18 yr) admitted to any ward or to ICU with AHRF attributable to ARDS or COVID-19 who received PP alongside with any oxygen delivery device, including simple oxygen therapy, HFNO, CPAP, and NIV	* PP in intubated patients *PP combined or mixed to lateral positioning *With a follow-up shorter than 7 days	Non-intubated, spontaneously breathing patients with AHRF attributable to ARDS or COVID-19(moderate-to-severe ARDS (PaO2/FiO2 < 200 mm Hg))	Adult patients (age ≥ 18 yrs old)	APP/ SP
E.X. Chua et al., 2021 [19]	*Observational (prospective or retrospective Cohort/case–control) *RCT *Examining PP versus SP * Adults (≥ 18 years old) diagnosed with COVID-19	*Case series *Case reports *Editorials	COVID-19 patients from general wards, emergency departments and ICUs	53–68 years old	PP/SP
Schmid et al., 2022 [20]	Studies investigating adult patients with severe respiratory failure due to COVID-19 infection according to the WHO clinical progression scale with the need for HFNO, NIV, or IMV	*Trials comparing different ventilator settings within HFNC or NIV, nor comparing simple oxygen insufflation or IMV to either HFNC or NIV *comparison of NIV vs. APP	Patients with severe RF (with the need for HFNO, NIV, or IMV) due to COVID-19(had to be confirmed by RT-PCR/clinically highly suspected) according to WHO clinical progression scale	47.4–74.8 years old	APP for as long as possible (≥ 16 h/day)/ unrestricted (self) positioning (standard care)
Pavlov et al., 2022 [21]	*Original research reports of COVID − 19 patients *Patients were treated with APP and/or HFNO or NIV or conventional oxygen therapy	*Languages other than English or Chinese *Study protocols, reviews, abstracts, editorials *Research on newborns or animals * < 3 cases reports	Patients with AHRF due to coronavirus disease 2019 (severe COVID-19 were treated with APP and/or HFNC or NIV or conventional oxygen therapy	NR	APP/standard care
Ponnapa Reddy et al., 2021 [22]	Studies on laboratory-confirmed SARS-2 hypoxemic adult patients requiring supplemental o2 who received PP and reported on Pao2/Fio2, Pao2,Spo2	*Narrative reviews *Studies that did not report oxygenation variables *Case reports or case series with ≤ five patients	In hypoxemic, nonintubated adult patients with coronavirus diseases 2019	Adult patients (≥ 18 old)	PP/preproning
Sryma PB et al., 2021 [23]	Studies on adults (at least five patients) with COVID-19 and AHRF, not requiring IMV, and employing awake PP as a therapeutic strategy	*Patient series(n < 5) as highly likely to present a biased outcome in the form of only favorable outcome reporting *Studies on PP during IMV as well as studies not reporting respiratory outcomes	Patients with COVID-19 AHRF confirmed COVID-19infection requiring oxygen supplementation or with room air saturation < 94%	58.42	There was no comparator group
M. T. Awad et al., 2021 [24]	RCTs, cohort studies, case–control studies, and case series	Animal studies, case reports, reviews, editorials, and letters to editors	COVID-19 patients with AHRF who were admitted to the hospital	NR	APP /non-APP
Beran et al., 2021 [25]	RCTs and observational studies that compared APP versus control group in non-intubated COVID-19 subjects and reported one of the following outcomes: TI, mortality, and length of hospital stay	*All the studies that did not report TI or mortality rates *Single-arm studies, case reports,reviews,commentaries, preprints (not peer-reviewed), and abstracts	Spontaneously breathing non-intubated patients with laboratory-confirmed COVID-19	56	APP /non-APP
Cardona et al., 2021 [26]	Studies of adult patients (age > 18 years) who underwent PP while awake and alert, prior to TI and IMV in meeting and poster abstracts, case series, retrospective and prospective studies, RCTs, quasi-randomized trials and eligible in-press articles	Studies that were not in the English language, included nonhuman subjects, did not present original data, or were case reports, included pediatric patients, failed to implement PP prior to TI, or did not report TI rates	Patients with COVID-19 requiring oxygen or NIV support who underwent APP, prior to TI and IMV	56.8	APP/standard care (only 3 study)
R. S. Cruz et al., 2021 [27]	RCTs that compared the use of APP with usual care in patient with AHRF due to COVID-19	NR	Covid-19 patients with ARHF because of ARDS	49–66	APP/usual care
Jie Li et al., 2022 [28]	RCTs and observational studies (with a control group) of APP in patients with COVID-19-related AHRF published in English	Trials that patients intubated before/at enrolment, pediatric patients, trials that did not include SP in control group	Non-intubated adult (≥ 18 years) patients hospitalized for COVID-19 COVID-19-related AHRF	Adult patients (≥ 18 old)	APP /SP
*Meta-analyses*					
W Tan et al., 2021 [29]	*Cohort studies or case series *Awake adult (18 years old) patients with AHRF or ARDS *PP combined with NIV *Outcomes including at least one of the following measures: aggregated mortality rate, TI rate, tolerability, prior to and following difference of (PaO2/FiO2)ratio,SpO2 and RR	*Patients who did not meet the screening criteria *Studies that were not in English or commentaries, reviews, duplicate publications from the same study *Data that could not be extracted by the statistical methods or non-targeted outcomes	Awake non-intubated adult patients with AHRF or ARDS	57 ± 11 years old	PP/SP
*Systematic reviews*					
G. T. Chilkoti et al., 2021 [30]	All kinds of publications that is case report, case series, editorials, letters, and reviews in addition to original articles providing evidence toward the efficacy of APP in the improvement of oxygenation in COVID-19 disease	NR	Patients with a validated diagnosis of COVID-19 and receiving PP	58	APP/not receiving prone oxygenation
S. Anand et al., 2020 [31]	All non-intubated COVID-19 patients, where diagnosis is made with RT-PCR,PP in comparison with SP and evaluation of patients requiring TI, improvement in oxygenation and mortality rates	Pregnant women were excluded	Awake and non-intubated patients having a confirmed diagnosis of COVID-19	≥ 50	PP/SP
Parashar et al., 2021 [32]	Published/in press peer-reviewed RCTs, case–control trials, and prospective/retrospective cohort studies assessing effectiveness of APP for NI patients diagnosed with COVID-19 confirmed by RT-PCR	Doubtful cases of COVID-19 without positive RT-PCR, intubated patients, or patients need to intubation, studies on animals, pregnant women, and children with COVID-19	Patients with COVID-19 as detected by RT-PCR in nasopharyngeal/oropharyngeal samples in non-ICU that were presented with hypoxia requiring supplemental O2 by nasalprong/facemask/HFNO/NIV	Adult patients (≥ 18 old)	APP/ Other standards of care
Alhazzani et al., 2021 [33]	Hospitalized NI patients with AHRF in PP in comparison with SP and evaluation of TI, survival, change in respiratory parameters, adverse events in observational or RCTs	Pregnant, non-awake, intubated patients	Severe and critical coronavirus disease 2019 in the ICU	Adult patients (≥ 18 old)	APP/no awake proning
Ko et al., 2021 [34]	PaO2/FiO2 ratio < 300 mmHg, mild respiratory alkalosis and no alkalemia, O2 supplement or noninvasive CPAP	ECMO	ICU admitted covid-19 patients	Adult patients (≥ 18 old)	*PP/standard care(only in 1 study) *Noncomperative in other studies
Sendero-vich H et al., 2022 [35]	Established treatment protocols using HCQ, RMD, FVP, RTV, MPV, DMS, BUD, TCZ, BMB, BNB, SVB, FLV, CP, PP, or anticoagulant therapy in older patients. primary outcomes: the improvement of COVID-19 symptoms, shortened duration of symptoms, decreased viral load, and CRP and in IL-6	NR	Geriatric patients with COVID-19	≥ 60	APP /SP

*ICU* Intensive Care Unit, *AHRF* Acute Hypoxemic Respiratory Failure, *ARDS* Acute Respiratory Distress Syndrome, *APP* Awake Prone Position, *PP* Prone Position, *SP* Supine Position, *CPAP* Continuous Positive Airway Pressure, *NIV* Non-Invasive Ventilation, *HFNO* High-Frequency Nasal Oxygen, *IMV* Invasive Mechanical Ventilation, *NI* Non-Intubated, *WHO* World Health Organization, *RCT* Randomized Controlled Trial, *PaO2/FiO2* Pressure of Arterial Oxygen to Fractional Inspired Oxygen Concentration, *PaO2* the partial pressure of oxygen in the arterial blood, *SpO2* Spot Oxygen Saturation, *TI* Tracheal Intubation, *RR* Respiratory Rate, *RT-PCR* Reverse transcription polymerase chain reaction, *NR* Not-Recorded, *ECMO* Extracorporeal Membrane Oxygenation

The investigated variables, software used for statistical analysis, primary and secondary outcomes, risk of bias assessment, publication bias, and quality of all included studies are shown in detail in Table 3.

**Table 3:** Endpoints and quality of included systematic reviews and meta-analysis studies

Author/year/reference	Variables	Software used for statistical analyses	Endpoint variables analyzed		Quality (ROB) Assessment	Quality of SR	Publication bias
			Primary outcomes	Secondary outcomes			
*Systematic reviews and meta-analyses*							
Fazzini et al., 2021 [18]	*PaO2/FiO2 after PP * Mortality *TI rate *Proning tolerating time	RevMan 5.4.1	Change in oxygenation pre and post PP reported as PaO2/FiO2	The rate of TI, mortality, ICU admission, limitations, and adverse events	*NOS *Cochrane risk-of-bias tool *GRADE	NR	High risk of bias
E.X. Chua et al., 2021 [19]	*PaO2/FiO2 ratio *Mortality *PaCO2(mmHg), SpO2(%) *TI incidence *Patients discharged	RevMan 5.3	PaO2/FiO2 ratio after PP and SP	SpO2, PaCO2, mortality rate, incidence of TI and number of patients discharged alive	GRADE	Very low	Low risk of bias
Schmid et al., 2022 [20]	*Mortality, TI and safety *All-cause mortality(28d) *TI within 28 d *Hospital length of stay (censored at 28 d) *Skin lesions within 28 d	NR	*All-cause mortality at d 28, 60, time-to-event, and at hospital discharge *Clinical status at d 28, 60, & up to longest FU *Quality of life *Serious adverse events	*Clinical status at d28, 60 & up to longest FU *ICU admission at d 28 *Hospitalization duration *Skin lesions	*GRADE *Statistical heterogeneity was defined as *p* < 0.1 for Chi^2^ test or or I^2^ ≥ 50%	Very low	NR
Pavlov et al., 2022 [21]	*In hospital TI *Mortality *Proning tolerating time *Respiratory parameters improvement	NR	In-hospital TI	In-hospital mortality	NR	Low**	High
Ponnapa Reddy et al., 2021 [22]	*PaO2/FiO2 *PaO2 *SpO2 *Secondary analysis based on ratio of PaO2/FiO2	Statistical software package Stata-Version 16	Change in oxygenation (Pao2/Fio2 ratio, Pao2, and Spo2) following PP	*Reduction in RR who underwent PP *Intubation *Mortality	*Newcastle–Ottawa Scale for cohort studies *Joanna Briggs Institute Critical Appraisal Checklist for case series	Low***	Egger’s regression test was used to examine publication bias
Sryma PB et al., 2021 [23]	*TI *Oxygenation parameter *PaO2: FiO2 *PaO2, SpO2, RR	STATA statistical analysis software	Need for TI and IMV	*Oxygenation *Mortality *Length of stay	The Qualsyst tool for quantitative studies	β	¥
M. T. Awad et al., 2021 [24]	* TI *Mortality		* TI *Mortality		NR	Moderate	NR
Beran et al., 2021 [25]	*TI *Mortality *Length of hospital stay	Review Manager 5.3	*Need for TI *Mortality	Length of hospital stay	Q statistic and I^2^ (*P* < 0.10 was considered significant)	Moderate or high quality	Low risk of bias^$$^
Cardona et al., 2021 [26]	*TI *Mortality	Software comprehensive Meta-Analysis	Rate of TI and IMV	*Rate of TI within 24 h of presentation *Any mortality rate	Standard and modified Newcastle–Ottawa Scales (NOS)	Moderate or high quality	NA
R. S. Cruz et al., 2021 [27]	*TI *Mortality *Gas exchange	RevMan 5.4	TI rate	*Mortality *Oxygenation	*Cochrane Collaboration tool *GRADE	Low to moderate quality ^**#**^	High risk of bias ^##^
Jie Li et al., 2022 [28]	Age,sex,BMI,comorbidity,baseline oxygenation, oxygen devices at enrolment, enrolment location, target and actual APP duration, and the use of corticosteroids	Meta package (version 5.0.1) in R (version 4.0.3)	Reported cumulative TI risk across RCTs	All-cause mortality, need for escalating respiratory support, duration of ICU and hospital stay, safety outcomes	GRADE The NOS was used to assess the risk of bias of observational studies, and full details	Very low	§
*Meta-analyses*							
W Tan et al., 2021 [29]	*TI *Mortality rate *Improvement in the paO2/fiO2 *Improvement in SpO2 *Changes in RR *Intolerance rate	R software (R version 4.0.2) and RevMan version 5.3	*TI rate *Mortality rate	*Improvement in the paO2/fiO2 *Improvement in SpO2 *Changes in RR *Intolerance rate	NOS	€	µ
*Systematic reviews*							
G. T. Chilkoti et al., 2021 [30]	*ICU or non-ICU setup *Mode of oxygen therapy *Outcomes: Change SpO2 after 5 min of PP rate of TI, mean PaO2, PaO2/FiO2, mortality *Duration of proning *Limitations	NA	*Change in median SpO2 after 5 min of PP *Rate of TI in patients who were proned *Mean PaO2 *PaO2/FiO2 *Mortality	*Duration of proning *Limitations	Cochrane Collaboration tool, namely ROBINS-I	###	Moderate to serious risk of bias
S.Anand et al., 2020 [31]	*Improvement in SpO2, PaO2/FiO2 ratio, PO2 and SaO2 *Intubation *Mortality	SPSS 20.0	*Intubation *Mortality rate	*Improvement in oxygenation	NR	Very low	NR
Parashar et al., 2021 [32]	*Assessing SpO2, ROX index, RR, PaO2, PaO2:FiO2, A-aO2 *Duration of proning *Needed TI and MV *Mortality rate	NR	Clinical improvement rate (in terms of improved oxygenation)	*Need for MV *Death rate *Length of hospital stay	NOS	Low	High
Alhazzani et al., 2021 [33]	*TI/IMV *Mortality—COVID-19 exclusively (assessed with: No control group) *Mortality COVID-19 ICU *Oxygenation	NR	1. Mortality 2. TI 3. Adverse events	NR	GRADE	Low	Low risk of bias^**∞**^
Ko et al., 2021 [34]	*Oxygenation *Need to TI *Mortality	NR	*28 d mortality *Organ support free days within 21 d	NR	GRADE	High	NR
Sendero-vich H et al., 2022 [35]	*Viral load *viral markers *Ventilator-free days *clinical improvement	NR	*Symptom improvement *COVID-19 symptoms shortened duration *Decreased viral load *CRP,IL-6		NR	NR	High

*PaO2/FiO2* Pressure of Arterial Oxygen to Fractional Inspired Oxygen Concentration, *PP* Prone Position, *TI* Tracheal Intubation, *PaCO2(mmHg)* measured the partial pressure of carbon dioxide in arterial blood, *SpO2(%)* Spot Oxygen Saturation, *Pao2* Partial Pressure of Oxygen, *RR* Respiratory Rate, *BMI* Body Mass Index, *APP* Awake Prone Position, *ICU* Intensive Care Unit, *ROX* respiratory rate oxygenation, *A-aO2* alveolar-arterial gradient, *MV* Mechanical Ventilation, *NR* not recorded, *NA* not applicable, *FU* Follow up, *ROB* Risk Of Bias, *NOS* The Newcastle–Ottawa Scale, *GRADE* Grades of Recommendation, Assessment, Development and Evaluation, *MMAT* Mixed Methods Appraisal Tool, *ROBINS* Risk of Bias In Non-randomized Studies—of Interventions, *RCT* Randomized Clinical Trial

^**^According to Cochran’s Q test, there was significant high heterogeneity among studies (*p* < 0.001) (more than 85%)

^***^High selectivity of subjects, application of PP inconsistency in published reports, outcomes heterogeneity based on available low-quality data

β Heterogeneity was tested using the chi-square test on Cochran’s Q statistic, which was calculated using H and I^2^ indices

¥ assessing by Cochran Q statistic and I^2^ test, publication bias assessment was done using the funnel plot, Begg’s test for publication bias

^$$^ Jadad composite scale for methodological quality of the RCTs, NOS for quality of the observational studies and funnel plot and quantitively using Egger’s regression analysis for publication bias represented low risk of bias

^#^Based on weighted kappa scores and Q statistic and the I^2^ statistic

^##^Downgrade risk of bias (performance bias), high risk of bias of all studies due to performance bias (blinding of patients and staff)

^§^No obvious risk of bias and publication bias was found among the included RCTs for the primary outcome. The summaries of the bias assessment of observational studies indicated potential reporting bias on mortality

€ Statistical heterogeneity assessment between studies was performed by Cochran’s Q test and reported with the I^2^ and Chi squared (χ^2^) statistics

µ The Egger’s test results (*p* < 0.001) and asymmetric funnel plot (Egger’s test funnel plots) suggested the presence of publication bias

^###^The Newcastle–Ottawa quality assessment scale (NOS) checklist statistical index (ranges from 0 to 100%) was used to measure the heterogeneity among the studies

∞The median NOS quality score for risk of bias was 3/9, with 11 studies scoring below 4

The extracted data related to selected parameters, including TI rate, mortality, and measures of PaO2/FiO2 and SpO2 for all included studies, are displayed in Table 4.

**Table 4:** Statistical data extraction table of included systematic review and meta-analysis studies

Author/year/reference	Effect model	Study statistical data	Tracheal intubation	Mortality	PaO2/FiO2 ratio pre/after PP	SpO2 (%)
Fazzini et al., 2021 [18]	Random effect model	Study (*N*) patient(*N*) APP (*N*)/ SP (*N*) APP (event *N*)/ SP (event *N*) RR (95% CI) MD (95% CI) *p*-value Heterogeneity (I2,p)	7 2095 824/1271 284/616 0.72 [0.43, 1.22] – 0.22 75%, 0.0006	6 2011 771/1240 150/312 0.57 [0.36, 0.93] – 0.02 51%, 0.07	4 189 (pre/after APP) 189 )pre/after APP( NR – − 23.10 [− 34.80, − 11.39] 0.0001 26%, 0.26	
E.X. Chua et al., 2021 [19]	Random effect model	Study (*N*) Patient (*N*) APP (*N*)/ SP (*N*) APP (event *N*)/ SP (event *N*) RR (95% CI) MD (95% CI) *p*-value Heterogeneity (I_2,_*p*)	5 626 223/403 80/143 1.20 [0.77, 1.86] – 0.42 25%, 0.25	4 427 168/259 20/72 0.35 [0.16, 0.75] – 0.007 28%, 0.22	5 423 172/251 NR – 68.81 [15.94, 121.69] 0.01 91%, < 0.00001	8 566 273/293 NR – 5.51 [3.17, 7.85] < 0.00001 95%, < 0.00001
Schmid et al., 2022 [20]	Random effect model	Study (*N*) Patient (*N*) APP (*N*)/ SP (*N*) APP (event *N*)/ SP (event *N*) RR (95% CI) MD (95% CI) *p*-value Heterogeneity (I_2,_*p*)	2 1196 600/596 NR 0.83 [0.71–0.96] – NR NR	2 1196 600/596 NR 1.08 [0.51–2.31] – NR NR		
Pavlov et al., 2022 [21]	Random effect model	Study (*N*) Patient (*N*) APP (*N*)/ SP (*N*) APP (event *N*)/ SP (event *N*) RR (95% CI) MD (95% CI) *p*-value Heterogeneity (I_2,_*p*)	25 1722 870/852 NR 0.27 [0.19, 0.37] – 0.71 88%, < 0.01	20 1528 767/761 NR 0.11 [0.06, 0.20] – 0.1 91%, < 0.01		
Ponnapa Reddy et al., 2021 [22]	Random effect model	Study (*N*) Patient (*N*) APP (*N*)/ SP (*N*) APP (event *N*)/ SP (event *N*) RR (95% CI) MD (95% CI) *p*-value Heterogeneity (I_2,_*p*)	15 697/699(pre/after APP) NR NR 0.24 [0.17–0.32] – NR 74.25%, 0.00	13 390 NR NR 0.13 [0.06, 0.19] – NR 83.55%, 0.00	22 695/695(pre/after APP) NR NR – 39.47 [24.85, 54.10] 0.001 99.67%, 0.00	12 38/38(pre/after APP) NR NR – 4.74[3.26, 6.23] 0.001 96.31%, 0.00
Sryma PB et al., 2021 [23]	Random effect model	Study (*N*) Patient (*N*) APP (*N*)/ SP (*N*) APP (event *N*)/ SP (event *N*) RR (95% CI) MD (95% CI) *p*-value Heterogeneity (I_2,_*p*)	15 334(pre/after APP) NR NR 0.25[0.16,0.34] – NR 62.16%, 0.00		4 91 (pre/after APP) NR NR – − 51.29 [− 88.67, − 13.91] 0.007 72%, 0.01	4 88 (pre/after APP) NR NR – − 5.39 [− 9.25, − 1.53] 0.006 97%, < 0.00001
M. T Awad et al., 2021 [24]	Random effect model	Study (*N*) Patient (*N*) APP (*N*)/ SP (*N*) APP (event *N*)/ SP (event *N*) RR (95% CI) MD (95% CI) *p*-value Heterogeneity (I_2,_*p*)	3 425 135/290 63/117 1.48 [0.75, 2.93] – 0.26 53.56%, 0.11	3 397 129/268 24/67 0.54 [0.22, 1.33] – 0.18 59.18%, 0.08		
Beran et al., 2021 [25]	Random effect model	Study (*N*) Patient (*N*) APP (*N*)/ SP (*N*) APP (event *N*)/ SP (event *N*) RR (95% CI) MD (95% CI) *p*-value Heterogeneity (I_2,_*p*)	14 3324 1495/1829 403/545 0.85 [0.66, 1.08] – 0.17 63%, 0.002	14 3242 1472/1770 263/400 0.68 [0.51, 0.90] – 0.008 52%, 0.02		
Cardona et al., 2021 [26]	Random effect model	Study (*N*) Patient (*N*) APP (*N*)/ SP (*N*) APP (event *N*)/ SP (event *N*) RR (95% CI) MD (95% CI) *p*-value Heterogeneity (I_2,_*p*)	18 364 NR NR 28.4% [20.1, 38.4] – 0.000 63%, 0.001	13 272 NR NR 14.1% [7.6, 24.7] – 0.000 62%, 0.002		
R. S. Cruz et al., 2021 [27]	Fixed effects (*I*_2_ < 20%) or random effects (*I*_2_ ≥ 20%)	Study (*N*) Patient (*N*) APP (*N*)/ SP (*N*) APP (event *N*)/ SP (event *N*) RR (95% CI) MD(95% CI) *p*-value Heterogeneity (I_2,_p)	7 1401 717/684 208/249 0.82 [0.71, 0.95] – 0.009 0%, 0.49	7 1401 717/684 133/144 0.90 [0.73, 1.11] – 0.34 16%, 0.31		
Jie Li et al., 2022 [28]	Random effects to heterogeneity and fixed effects to evaluate small studies influence	(a) Study (*N*) Patient (*N*) APP (*N*)/ SP (*N*) APP (event *N*)/ SP (event *N*) RR (95% CI) MD(95% CI) *p*-value Heterogeneity (I_2,_*p*)	*RCTs* 10 1985 1013/972 216/255 0.84 [0·72–0·97] – NR 0%, 0.96	*RCTs* 10 1985 1013/972 135/143 1·00 [0·70 − 1·44] – NR 0%, 0.79		
		(b) Study (*N*) Patient (*N*) APP (*N*)/ SP (*N*) APP (event *N*)/ SP (event *N*) RR (95% CI) MD(95% CI) *p*-value Heterogeneity (I2,*p*)	*Non-RCTs* 18 2506 1066/1440 254/626 0.62 [0.47, 0.83] – NR 65%, < 0.01	*Non-RCTs* 17 2501 1080/1421 187/433 0.56 [0.48–0.65] – NR 0%, 0.49		
W. Tan et al., 2021 [29]	Random effect model	Study (*N*) Patient(*N*) APP (*N*)/ SP (*N*) APP (event *N*)/ SP (event *N*) RR (95% CI) MD(95% CI) *p*-value Heterogeneity (I_2,_*p*)	11 195 NR NR 0.32 [0.23, 0.43] – NR 36%	9 138 NR NR 0.03[0.00, 0.07] – NR 0%	4 61 (pre/after APP) NR NR – 52.06 [5.36, 98.76] 0.03 81%, 0.005	5 112 (pre/after APP) NR NR – 5.23 [1.25, 9.22] 0.01 98%, < 0.00001

*PaO2/FiO2* Pressure of Arterial Oxygen to Fractional Inspired Oxygen Concentration, *SpO2* Spot Oxygen Saturation, *PaCO2* measured the partial pressure of carbon dioxide in arterial blood, *N* number, *CI* Confidence Interval, *RR* Respiratory Rate, *MD* Mean Difference, *RCT* Randomized Clinical Trial, *PP* Prone Position, *pre/after APP* before and after awake prone positioning, *APP (N)/ SP (N)* number of patients in awake prone position group/number of patient in supine group, *APP (event N)/ SP (event N)* number of event(intubation or mortality) in awake prone positioning group/ number of event(intubation or mortality) in supine position group

A summary of the results and conclusions of all included studies are displayed in Table 2 (Additional file 2: Table S2).

### Tracheal intubation rates

**Fig. 2:**
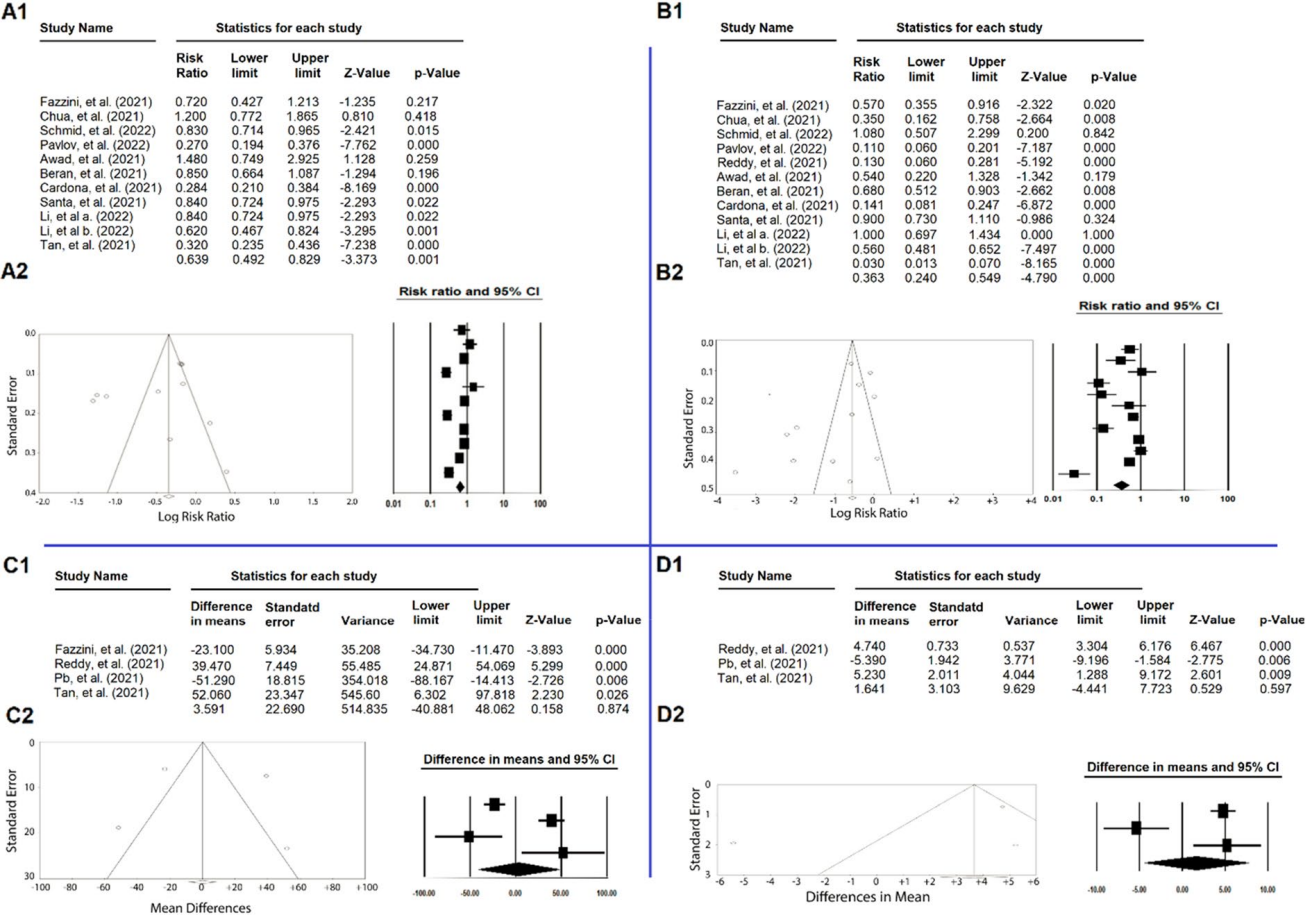
**A1** The forest plot shows the Odds ratios of the individual effect of APP versus SP on the tracheal intubation of awake non-intubated patients with COVID-19 pneumonia. **A2** Funnel plot standard error by log risk ratio to assess diffusion bias related to the effect of PP versus SP on the tracheal intubation of awake non-intubated patients with COVID-19 pneumonia. **B1** The forest plot shows the Odds ratios of the individual effect of APP versus SP on the mortality of awake non-intubated patients with COVID-19 pneumonia. **B2** Funnel plot standard error by log risk ratio to assess diffusion bias related to the effect of PP versus SP on the mortality rate of awake non-intubated patients with COVID-19 pneumonia. **C1** The forest plot shows differences in means of the effect of the PaO2/FiO2 of the awake non-intubated patients with COVID-19 pneumonia before and after PP. **C2** Funnel plot diagram to assess diffusion bias related to PaO2/FiO2 of the awake non-intubated patients with COVID-19 pneumonia before and after PP. **D1** The forest plot shows differences in the means of the effect of the SpO2 (%) of the awake non-intubated patients with COVID-19 pneumonia before and after PP. **D2** Funnel plot diagram to assess diffusion bias related to the SpO2 (%) of the awake non-intubated patients with COVID-19 pneumonia before and after PP

Eleven studies were included in this study. The heterogeneity between the studies was significant (*Q* = 119.4, *P* < 0.001, *I*^2^ = 91.6%). To estimate the pooled relative risk between the two groups, the OR values for each included study were entered into the data analysis. Figure 2(A1) illustrates the forest plot for the pooled effect size of the selected studies. Based on the random effect model, the pooled relative risk to assess the effect of the awake prone position in non-intubated patients with hypoxemic respiratory failure caused by severe COVID-19 on tracheal intubation rate was equal to 0.639 units, which was statistically significant (Pooled R.R = 0.639, %95 CI = (0.492, 0.829), *P*-value = 0.001). The publication bias was not significant as tested by Begg’s test (*P*-value = 0.350) (Fig. 2A2).

### Mortality rate

Twelve studies were included in this study. The heterogeneity between the studies was significant (*Q* = 147.3, *P* < 0.001, *I*^2^ = 92.5%). To estimate the pooled relative risk between the two groups, the relative risk associated with the study's purpose for each included study was entered into the data analysis. Figure 2B1 illustrates the forest plot for the pooled effect size of the selected studies. Based on the random effect model, the pooled relative risk to assess the effect of the awake prone position in non-intubated patients with hypoxemic respiratory failure caused by severe COVID-19 on mortality rate was equal to 0.363 units, which was statistically significant (pooled relative risk = 0.363 [0.240, 0.549], *P*-value < 0.001). There was no perceived publication bias despite an asymmetric funnel plot (*P*-value = 0.115) (Fig. 2B2).

### PaO2/FiO2

Four studies that reported on this outcome variable were included in this analysis. The heterogeneity between the studies was significant (*Q* = 48.1, *P* < 0.001, *I*^2^ = 93.8%). To estimate the pooled mean differences, the MD values from the individual meta-analyses before and after the application of PP in awake patients were entered for a pooled analysis. Figure 2C1 illustrates the forest plot for the pooled effect size of the selected studies. Based on the random effect model, the pooled mean differences to assess the effect of the awake prone position in non-intubated patients with hypoxemic respiratory failure caused by severe COVID-19 on the PaO2/FiO2 ratio was equal to 3.59 units [− 40.881, 48.062], *P*-value = 0.874, which wasn’t statistically significant. The funnel plot and Begg’s test were used to evaluate publication bias, and the results indicated that publication bias with a *P*-value = 0.734 was not significant (Fig. 2C2).

### Oxygen saturation

Three studies were included in this study. The heterogeneity between the studies was significant (*Q* = 24.5, *P* < 0.001, *I*^2^ = 91.8%). To estimate the pooled mean differences, the MD values before and after the PP in awake COVID-19 patients for the included studies were entered into the analysis. Figure 2D1 illustrates the forest plot for the pooled effect size of the selected studies. Based on the random effect model, the pooled mean difference to assess the effect of the PP in awake non-intubated patients with hypoxemic respiratory failure caused by severe COVID-19 on SpO2 (%) was equal to 1.64 units [− 4.441, 7.723]; with a non-significant *P*-value of 0.597. Additionally, there was no evidence of publication bias (*P* = 0.999) (Fig. 2D2).

## Discussion

The use of non-drug approaches, such as changing the patient's position to prone, can significantly help to improve oxygenation parameters in patients with ARDS. In hypoxemic COVID-19 patients, the APP method is used to avoid tracheal intubation, but its effectiveness is unclear. The early and timely use of the PP method in awake, non-intubated patients is one of the strategies to reduce ICU transfer and endotracheal intubation during the COVID-19 pandemic, following health system control and optimal ventilator use and reducing the economic burden. In this field, twelve systematic review and meta-analysis studies and six systematic review studies have been conducted [22–39]. This Review of Reviews will discuss a comprehensive review of the studies above.

In a case–control study of 29 patients with severe COVID-19 pneumonia, PP was started within the first 12 h of hospitalization, 18 patients tolerated it for at least 10 h per day, and 11 patients had no issues with it. According to the findings of this study, oxygenation improves in COVID-19-induced ARDS with early PP for 10 h per day [40]. The PaO2/FiO2 ratio in non-invasively ventilated patients such as the prone position is much higher than other methods of ventilatory support [41]. Burton-Papp et al. discovered a significant role for the combination of NIV and PP in improving oxygenation in a study of 20 conscious patients with COVID-19 pneumonia by measuring the PaO2/FiO2 ratio [42]. The standard treatment method was compared to the PP method in 60 non-intubated awake patients in a multicenter RCT in patients with acute respiratory failure secondary to COVID-19 infection. The results showed that awake PP was functional and valuable in improving the oxygenation [43]. In a cohort study of 25 patients with severe respiratory failure due to COVID-19 (12 intubated patients and 13 conscious patients), patients with SaO2 > 95% improved their oxygenation parameters. They were less likely to require intubation after one hour of PP [44].

The effects of improving oxygenation were permanent even after repositioning in the prospective cohort study by Coppo et al. on the physiological impact of PP in 56 patients with COVID-19 with severe respiratory failure [45]. According to previous studies, the rate of intubation did not decrease in a randomized multicenter clinical trial in patients with moderate to severe respiratory failure due to COVID-19 treated with high flow nasal oxygen or NIV and a PaO2/FiO2 ratio ≤ 200 by randomly dividing them into two groups of 16 h of PP per day or standard treatment [46].

In a retrospective cohort study of 97 hospitalized patients with COVID-19, it was discovered that measuring the respiratory oxygenation (ROX) index and the PaO2/FiO2 (partial pressure of oxygen/fraction of inspired oxygen) ratio in awake patients on PP can predict the time required for intubation [47]. Evidence suggests that the benefits of PP decline after significant disease progression and the onset of pulmonary fibrosis, emphasizing the significance of early PP initiation [48]. One study found that the prophylactic beginning of PP in COVID-19 patients significantly increased oxygenation [5]. The positive effects of PP outweighed its negative impact in a scoping review of its effects in patients with COVID-19 pneumonia [49]. The advantage of using this method in non-intubated, conscious patients who are not intubated is that complications like endotracheal tube displacement and dislocation in PP are not present in these patients [50]. The improvement in SpO2/FiO2 in the study of the effect of serial APP on oxygenation of non-intubated patients admitted to the ICU is usually temporary and often occurs in the first episode of this procedure, which may be the reason for not reporting the effect of APP on mortality in recent studies [51, 52]. In a narrative review, the APP method is recommended in critically ill patients with hypoxemic respiratory failure due to moderate to severe COVID-19, as long as there is no delay in mechanical ventilation [53].

The limitation of the study was the identification of systematic review studies conducted prone position on intubated patients from conscious patients. Especially, in some studies, PP was performed on both conscious and intubated patients, which made it difficult to extract the results related to conscious patients.

Each of the 18 review studies recommended using APP in COVID-19 pneumonia. To reach a more definitive conclusion about the effect of APP on COVID-19 pneumonia, the results of this Review of Reviews, which is a comprehensive study of all previous reviews and meta-analyses, show that APP has a significant effect on reducing tracheal intubation and mortality, but not on PaO2/FiO2 and SpO2 (%). According to the results of 18 systematic reviews on 22,562 patients, the results of this Review of Reviews can be generalized, and APP can be recommended with certainty in the treatment of conscious and non-intubated patients with COVID-19 pneumonia to reduce mortality and tracheal intubation.

## Conclusion

With more confidence, the results of this Review of Reviews showed the influential role of APP in reducing mortality and intubation, which can be recommended in the treatment period of conscious patients with COVID-19 pneumonia. It is advisable to consider the use of prone positioning in conscious patients with COVID-19 pneumonia in the ICU as a means to decrease mortality rates and the need for intubation. 

### Supplementary Information

**Additional file 1: Table S1.** Search Strategy Using database-appropriate syntax with parentheses, Boolean operators, and field codes.

**Additional file 2: Table S2.** Results and conclusions of the included systematic reviews and meta-analyses.

## Data Availability

All data generated or analyzed during this study are included in this published article (and its additional information files).

## References

[1] Shahsavarinia K, Ghojazadeh M, Ghabousian A, Hatefnia F, Soleimanpour M, Soleimanpour H (2021). An umbrella review of clinical efficacy and adverse cardiac events associated with hydroxychloroquine or chloroquine with or without azithromycin in patients with COVID-19. Anesth Pain Med.

[2] Shadvar K, Tagizadiyeh A, Gamari AA, Soleimanpour H, Mahmoodpoor A (2021). Hemoperfusion as a potential treatment for critically ill COVID-19 patients with cytokine storm. Blood Purif.

[3] Shahsavarinia K, Ghojazadeh M, Sanaie S, Vahedi L, Ahmadpour M, Mahmoodpoor A (2021). Clinical efficacy of hydroxychloroquine or chloroquine in patients with COVID-19: an umbrella review. Pharm Sci.

[4] Bagi HM, Soleimanpour M, Abdollahi F, Soleimanpour H (2021). Evaluation of clinical outcomes of patients with mild symptoms of coronavirus disease 2019 (COVID-19) discharged from the emergency department. PLoS ONE.

[5] Mahmoodpoor A, Shadvar K, Ghamari AA, Mohammadzadeh Lameh M, Asghari Ardebili R, Hamidi M (2020). Management of critically ill patients with COVID-19: what we learned and what we do. Anesth Pain Med.

[6] Tekantapeh ST, Mikaeili H, Suleimanpour H (2021). The role of respiratory system surface area and ventilation volume in severity and mortality of COVID-19 infection. Front Emerg Med.

[7] Bhurayanontachai R (2021). Mechanical ventilator support and prone positioning in COVID-19 related pneumonia: mechanical ventilator support in COVID-19. Clin Crit Care.

[8] Ng Z, Tay WC, Ho CHB (2020). Awake prone positioning for non-intubated oxygen dependent COVID-19 pneumonia patients. Eur Respir J.

[9] Seckel MA (2021). Awake self-prone positioning and the evidence. Crit Care Nurse.

[10] Sodhi K, Chanchalani G (2020). Awake proning: current evidence and practical considerations. Indian J Crit Care Med.

[11] Touchon F, Trigui Y, Prud'homme E, Lefebvre L, Giraud A, Dols AM (2021). Awake prone positioning for hypoxaemic respiratory failure: past, COVID-19 and perspectives. Eur Respir Rev.

[12] Taboada M, Bermudez A, Perez M, Campana O (2020). Supine versus prone positioning in COVID-19 Pneumonia: comment. Anesthesiology.

[13] Tahsini Tekantapeh S, Ghojazadeh M, Ghamari AA, Mohammadi A, Soleimanpour H (2022). Therapeutic and anti-inflammatory effects of baricitinib on mortality, ICU transfer, clinical improvement, and CRS-related laboratory parameters of hospitalized patients with moderate to severe COVID-19 pneumonia: a systematic review and meta-analysis. Expert Rev Respir Med.

[14] Hasan N (2021). Awake prone positioning in Sars-CoV-2 (COVID-19) non-intubated patients with acute respiratory failure in adult population: a literature review. Med Sci Discov.

[15] Koeckerling D, Barker J, Mudalige NL, Oyefeso O, Pan D, Pareek M (2020). Awake prone positioning in COVID-19. Thorax.

[16] Stilma W, Akerman E, Artigas A, Bentley A, Bos LD, Bosman TJC (2021). Awake proning as an adjunctive therapy for refractory hypoxemia in non-intubated patients with COVID-19 acute respiratory failure: guidance from an international group of healthcare workers. Am J Trop Med Hyg.

[17] Khan S, Choudry E, Mahmood SU, Mulla AY, Mehwish S (2020). Awake proning: a necessary evil during the COVID-19 pandemic. Cureus.

[18] Bentley SK, Iavicoli L, Cherkas D, Lane R, Wang E, Atienza M (2020). Guidance and patient instructions for proning and repositioning of awake, nonintubated COVID-19 patients. Acad Emerg Med.

[19] Prasad M, Visrodia K (2020). Should I prone non-ventilated awake patients with COVID-19?. Cleve Clin J Med.

[20] Fiorentino G, Esquinas AM, Piervincenzi E (2021). Insights about prone and lateral positioning in spontaneously breathing patients with COVID-19 pneumonia undergoing noninvasive helmet CPAP treatment. Chest.

[21] Nauka PC, Chekuri S, Aboodi M, Hope AA, Gong MN, Chen JT (2021). A case-control study of prone positioning in awake and nonintubated hospitalized coronavirus disease 2019 patients. Crit Care Explor.

[22] Chua EX, Zahir S, Ng KT, Teoh WY, Hasan MS, Ruslan SRB (2021). Effect of prone versus supine position in COVID-19 patients: a systematic review and meta-analysis. J Clin Anesth.

[23] Fazzini B, Page A, Pearse R, Puthucheary Z (2022). Prone positioning for non-intubated spontaneously breathing patients with acute hypoxaemic respiratory failure: a systematic review and meta-analysis. Br J Anaesth.

[24] Ponnapa Reddy M, Subramaniam A, Afroz A, Billah B, Lim ZJ, Zubarev A (2021). Prone positioning of nonintubated patients with coronavirus disease 2019—a systematic review and meta-analysis. Crit Care Med.

[25] Schmid B, Griesel M, Fischer AL, Romero CS, Metzendorf MI, Weibel S (2022). Awake prone positioning, high-flow nasal oxygen and non-invasive ventilation as non-invasive respiratory strategies in COVID-19 acute respiratory failure: a systematic review and meta-analysis. J Clin Med.

[26] Pavlov I, He H, McNicholas B, Perez Y, Tavernier E, Trump MW, et al. Awake prone positioning in non-intubated patients with acute hypoxemic respiratory failure due to COVID-19. Respir Care. 2021. 10.4187/respcare.09191 34234032

[27] Pb S, Mittal S, Madan K, Mohan A, Tiwari P, Hadda V (2021). Awake prone positioning in non-intubated patients for the management of hypoxemia in COVID-19: a systematic review and meta-analysis. Monaldi Arch Chest Dis.

[28] Ni Z, Wang K, Wang T, Ni Y, Huang W, Zhu P (2020). Efficacy of early prone or lateral positioning in patients with severe COVID-19: a single-center prospective cohort. Precis Clin Med.

[29] Beran A, Mhanna M, Srour O, Ayesh H, Sajdeya O, Ghazaleh S (2022). Effect of prone positioning on clinical outcomes of non-intubated subjects with COVID-19. Respir Care.

[30] Cardona S, Downing J, Alfalasi R, Bzhilyanskaya V, Milzman D, Rehan M (2021). Intubation rate of patients with hypoxia due to COVID-19 treated with awake proning: a meta-analysis. Am J Emerg Med.

[31] Li J, Luo J, Pavlov I, Perez Y, Tan W, Roca O (2022). Awake prone positioning for non-intubated patients with COVID-19-related acute hypoxaemic respiratory failure: a systematic review and meta-analysis. Lancet Respir Med.

[32] Santa Cruz R, Irrazabal C, Gonzalez L, Geloso A, Nunez C, Cornejo R (2022). Analytic review and meta-analysis of awake prone positioning in patients with COVID-19. Med Intensiva.

[33] Tan W, Xu DY, Xu MJ, Wang ZF, Dai B, Li LL (2021). The efficacy and tolerance of prone positioning in non-intubation patients with acute hypoxemic respiratory failure and ARDS: a meta-analysis. Ther Adv Respir Dis.

[34] Alhazzani W, Evans L, Alshamsi F, Moller MH, Ostermann M, Prescott HC (2021). Surviving sepsis campaign guidelines on the management of adults with coronavirus disease 2019 (COVID-19) in the ICU: first update. Crit Care Med.

[35] Anand S, Baishya M, Singh A, Khanna P (2021). Effect of awake prone positioning in COVID-19 patients—a systematic review. Trends Anaesth Crit Care.

[36] Chilkoti GT, Mohta M, Saxena AK, Ahmad Z, Sharma CS (2021). Awake prone positioning in the management of COVID-19 pneumonia: a systematic review. Indian J Crit Care Med.

[37] Ko HK, Yu WK, Pan SW, Chen WC, Yang KY, Lin YT (2022). Consensus statement and recommendations on the treatment of COVID-19: 2021 update. J Chin Med Assoc.

[38] Parashar S, Karthik AR, Gupta R, Malviya D (2021). Awake proning for nonintubated adult hypoxic patients with COVID-19: a systematic review of the published evidence. Indian J Crit Care Med.

[39] Senderovich H, Vinoraj D, Stever M, Waicus S (2022). Efficacy of COVID-19 treatments among geriatric patients: a systematic review. Ther Adv Infect Dis.

[40] Simioli F, Annunziata A, Langella G, Martino M, Musella S, Fiorentino G (2021). Early prone positioning and non-invasive ventilation in a critical COVID-19 subset. A single centre experience in southern Italy. Turk Thorac J..

[41] Capsoni N, Privitera D, Airoldi C, Gheda S, Mazzone A, Terranova G, Galbiati F, Bellone A (2023). Evaluation of PaCO 2 trend in COVID-19 patients undergoing helmet CPAP in the emergency department. Emerg Care J..

[42] Burton-Papp HC, Jackson AIR, Beecham R, Ferrari M, Nasim-Mohi M, Grocott MPW (2020). Conscious prone positioning during non-invasive ventilation in COVID-19 patients: experience from a single centre. F1000Res.

[43] Jayakumar D, Ramachandran Dnb P, Rabindrarajan Dnb E, Vijayaraghavan Md BKT, Ramakrishnan Ab N, Venkataraman AR (2021). Standard care versus awake prone position in adult nonintubated patients with acute hypoxemic respiratory failure secondary to COVID-19 infection—a multicenter feasibility randomized controlled trial. J Intensive Care Med.

[44] Thompson AE, Ranard BL, Wei Y, Jelic S (2020). Prone positioning in awake, nonintubated patients with COVID-19 hypoxemic respiratory failure. JAMA Intern Med.

[45] Coppo A, Bellani G, Winterton D, Di Pierro M, Soria A, Faverio P (2020). Feasibility and physiological effects of prone positioning in non-intubated patients with acute respiratory failure due to COVID-19 (PRON-COVID): a prospective cohort study. Lancet Respir Med.

[46] Rosen J, von Oelreich E, Fors D, Jonsson Fagerlund M, Taxbro K, Skorup P (2021). Awake prone positioning in patients with hypoxemic respiratory failure due to COVID-19: the PROFLO multicenter randomized clinical trial. Crit Care.

[47] Downing J, Cardona S, Alfalasi R, Shadman S, Dhahri A, Paudel R (2021). Predictors of intubation in COVID-19 patients undergoing awake proning in the emergency department. Am J Emerg Med.

[48] Adeola JO, Patel S, Goné EN, Tewfik G (2021). A quick review on the multisystem effects of prone position in acute respiratory distress syndrome (ARDS) including COVID-19. Clin Med Insights Circ Respir Pulm Med.

[49] Araújo MS, Santos M, Silva CJA, Menezes RMP, Feijão AR, Medeiros SM (2021). Prone positioning as an emerging tool in the care provided to patients infected with COVID-19: a scoping review. Rev Lat Am Enfermagem.

[50] Venus K, Munshi L, Fralick M (2020). Prone positioning for patients with hypoxic respiratory failure related to COVID-19. CMAJ.

[51] Barker J, Pan D, Koeckerling D, Baldwin AJ, West R (2022). Effect of serial awake prone positioning on oxygenation in patients admitted to intensive care with COVID-19. Postgrad Med J.

[52] Ehrmann S, Li J, Ibarra-Estrada M, Perez Y, Pavlov I, McNicholas B, Roca O, Mirza S, Vines D, Garcia-Salcido R, Aguirre-Avalos G (2021). Awake prone positioning for COVID-19 acute hypoxaemic respiratory failure: a randomised, controlled, multinational, open-label meta-trial. Lancet Respir Med.

[53] Qadri SK, Ng P, Toh TSW, Loh SW, Tan HL, Lin CB (2020). Critically ill patients with COVID-19: a narrative review on prone position. Pulm Ther.

