# Supplementary material for: Prone positioning effect on tracheal intubation rate, mortality and oxygenation parameters in awake non-intubated severe COVID-19-induced respiratory failure: a review of reviews
Source: Eur J Med Res. 2024 Jan 20;29:63. doi: 10.1186/s40001-024-01661-6 (PMC10799467; doi:10.1186/s40001-024-01661-6)
Supplement: Supplementary file 1 — Additional file 1: Table S1. Search Strategy Using database-appropriate syntax with parentheses, Boolean operators, and field codes. [file 40001_2024_1661_MOESM1_ESM.docx]

**Table S1.** Search Strategy Using database-appropriate syntax with parentheses, Boolean operators, and field codes.

| Database | Syntax | Records Number |
| --- | --- | --- |
| PubMed | ("COVID-19 pandemic"[MeSH Terms]) OR ("COVID-19 pandemic"[Other Term] OR ("COVID-19 pandemic"[Title/Abstract])) OR ("COVID19 pandemic"[MeSH Terms])) OR ("COVID19 pandemic"[Other Term])) OR ("COVID19 pandemic"[Title/Abstract])) OR ("COVID-19 pandemics"[MeSH Terms])) OR ("COVID-19 pandemics"[Other Term])) OR ("COVID-19 pandemics"[Title/Abstract])) OR ("Severe Acute Respiratory Syndrome Coronavirus 2 infection"[MeSH Terms])) OR ("Severe Acute Respiratory Syndrome Coronavirus 2 infection"[Other Term])) OR ("Severe Acute Respiratory Syndrome Coronavirus 2 infection"[Title/Abstract])) OR ("SARS Coronavirus 2 Infection"[MeSH Terms])) OR ("SARS Coronavirus 2 Infection"[Other Term])) OR ("SARS Coronavirus 2 Infection"[Title/Abstract])) OR ("COVID-19 Virus Disease"[MeSH Terms])) OR ("COVID-19 Virus Disease"[Other Term])) OR ("COVID-19 Virus Disease"[Title/Abstract])) OR ("COVID19 Virus Disease"[MeSH Terms])) OR ("COVID19 Virus Disease"[Other Term])) OR ("COVID19 Virus Disease"[Title/Abstract])) OR ("COVID-19 Virus Diseases"[MeSH Terms])) OR ("COVID-19 Virus Diseases"[Other Term])) OR ("COVID-19 Virus Diseases"[Title/Abstract])) OR ("2019-nCoV Infection"[MeSH Terms])) OR ("2019-nCoV Infection"[Other Term])) OR ("2019-nCoV Infection"[Title/Abstract])) OR ("2019-nCoV Infections"[MeSH Terms])) OR ("2019-nCoV Infections"[Other Term])) OR ("2019-nCoV Infections"[Title/Abstract])) OR ("COVID19"[MeSH Terms])) OR ("COVID19"[Other Term])) OR ("COVID19"[Title/Abstract])) OR ("Coronavirus Disease 2019"[MeSH Terms])) OR ("Coronavirus Disease 2019"[Other Term])) OR ("Coronavirus Disease 2019"[Title/Abstract])) OR ("Coronavirus Disease-19"[MeSH Terms])) OR ("Coronavirus Disease-19"[Other Term])) OR ("Coronavirus Disease-19"[Title/Abstract])) OR ("Coronavirus Disease 19"[MeSH Terms])) OR ("Coronavirus Disease 19"[Other Term])) OR ("Coronavirus Disease 19"[Title/Abstract])) OR ("SARS-CoV-2 Infection"[MeSH Terms])) OR ("SARS-CoV-2 Infection"[Other Term])) OR ("SARS-CoV-2 Infection"[Title/Abstract])) OR ("SARS-CoV-2 Infections"[MeSH Terms])) OR ("SARS-CoV-2 Infections"[Other Term])) OR ("SARS-CoV-2 Infections"[Title/Abstract])) OR ("SARS CoV 2 Infection"[MeSH Terms])) OR ("SARS CoV 2 Infection"[Other Term])) OR ("SARS CoV 2 Infection"[Title/Abstract])) OR ("2019 Novel Coronavirus Disease"[MeSH Terms])) OR ("2019 Novel Coronavirus Disease"[Other Term])) OR ("2019 Novel Coronavirus Disease"[Title/Abstract])) OR ("2019 Novel Coronavirus Infection"[MeSH Terms])) OR ("2019 Novel Coronavirus Infection"[Other Term])) OR ("2019 Novel Coronavirus Infection"[Title/Abstract])) OR ("2019-nCoV Disease"[MeSH Terms])) OR ("2019-nCoV Disease"[Other Term])) OR ("2019-nCoV Disease"[Title/Abstract])) OR ("2019-nCoV Diseases"[MeSH Terms])) OR ("2019-nCoV Diseases"[Other Term])) OR ("2019-nCoV Diseases"[Title/Abstract])) OR ("2019 nCoV Disease"[MeSH Terms])) OR ("2019 nCoV Disease"[Other Term])) OR ("2019 nCoV Disease"[Title/Abstract])) OR ("COVID-19 Virus Infection"[MeSH Terms])) OR ("COVID-19 Virus Infection"[Other Term])) OR ("COVID-19 Virus Infection"[Title/Abstract])) OR ("COVID-19 Virus Infections"[MeSH Terms])) OR ("COVID-19 Virus Infections"[Other Term])) OR ("COVID-19 Virus Infections"[Title/Abstract])) OR ("SARS Coronavirus 2"[Title/Abstract])) OR ("SARS Coronavirus 2"[MeSH Terms])) OR ("SARS Coronavirus 2"[Other Term])) OR ("Coronavirus Disease 2019 Virus"[MeSH Terms])) OR ("Coronavirus Disease 2019 Virus"[Other Term])) OR ("Coronavirus Disease 2019 Virus"[Title/Abstract])) OR ("2019 Novel Coronavirus"[MeSH Terms])) OR ("2019 Novel Coronavirus"[Other Term])) OR ("2019 Novel Coronavirus"[Title/Abstract])) OR ("2019 Novel Coronaviruses"[MeSH Terms])) OR ("2019 Novel Coronaviruses"[Other Term])) OR ("2019 Novel Coronaviruses"[Title/Abstract])) OR ("Severe Acute Respiratory Syndrome Coronavirus 2"[MeSH Terms])) OR ("Severe Acute Respiratory Syndrome Coronavirus 2"[Other Term])) OR ("Severe Acute Respiratory Syndrome Coronavirus 2"[Title/Abstract])) OR ("Wuhan Seafood Market Pneumonia Virus"[MeSH Terms])) OR ("Wuhan Seafood Market Pneumonia Virus"[Other Term])) OR ("Wuhan Seafood Market Pneumonia Virus"[Title/Abstract])) OR ("SARS- CoV-2 Virus"[MeSH Terms])) OR ("SARS- CoV-2 Virus"[Other Term])) OR ("SARS- CoV-2 Virus"[Title/Abstract])) OR ("SARS- CoV-2 Viruses"[MeSH Terms])) OR ("SARS- CoV-2 Viruses"[Other Term])) OR ("SARS- CoV-2 Viruses"[Title/Abstract])) OR ("SARS CoV 2 Virus"[MeSH Terms])) OR ("SARS CoV 2 Virus"[Other Term])) OR ("SARS CoV 2 Virus"[Title/Abstract])) OR ("2019-nCoV"[MeSH Terms])) OR ("2019-nCoV"[Other Term])) OR ("2019-nCoV"[Title/Abstract])) OR ("COVID-19 Virus"[MeSH Terms])) OR ("COVID-19 Virus"[Other Term])) OR ("COVID-19 Virus"[Title/Abstract])) OR ("COVID 19 Virus"[MeSH Terms])) OR ("COVID 19 Virus"[Other Term])) OR ("COVID 19 Virus"[Title/Abstract])) OR ("COVID-19 Viruses"[MeSH Terms])) OR ("COVID-19 Viruses"[Other Term])) OR ("COVID-19 Viruses"[Title/Abstract])) OR ("Wuhan Coronavirus"[MeSH Terms])) OR ("Wuhan Coronavirus"[Other Term])) OR ("Wuhan Coronavirus"[Title/Abstract])) OR ("COVID 19 Virus"[MeSH Terms])) OR ("COVID 19 Virus"[Other Term])) OR ("COVID 19 Virus"[Title/Abstract])) OR ("COVID 19 Viruses"[MeSH Terms])) OR ("COVID 19 Viruses"[Other Term])) OR ("COVID 19 Viruses"[Title/Abstract])) AND (((((((((((((((((((((((((((((((((((("awake proning"[MeSH Terms]) OR ("awake proning"[Other Term])) OR ("awake proning"[Title/Abstract])) OR ("prone"[MeSH Terms])) OR ("prone"[Other Term])) OR ("prone"[Title/Abstract])) OR ("prone position"[MeSH Terms])) OR ("prone position"[Other Term])) OR ("prone position"[Title/Abstract])) OR ("proning"[MeSH Terms])) OR ("proning"[Other Term])) OR ("proning"[Title/Abstract])) OR ("prone positioning"[MeSH Terms])) OR ("prone positioning"[Other Term])) OR ("prone positioning"[Title/Abstract])) OR ("prone oxygenation"[MeSH Terms])) OR ("prone oxygenation"[Other Term])) OR ("prone oxygenation"[Title/Abstract])) OR ("awake prone position"[MeSH Terms])) OR ("awake prone position"[Other Term])) OR ("awake prone position"[Title/Abstract])) OR ("self proning"[MeSH Terms])) OR ("self proning"[Other Term])) OR ("self proning"[Title/Abstract])) OR ("self-proning"[MeSH Terms])) OR ("self-proning"[Other Term])) OR ("self-proning"[Title/Abstract])) OR ("early awake prone"[MeSH Terms])) OR ("early awake prone"[Other Term])) OR ("early awake prone"[Title/Abstract])) OR ("awake prone positioning"[MeSH Terms])) OR ("awake prone positioning"[Other Term])) OR ("awake prone positioning"[Title/Abstract])) OR ("non-intubated"[MeSH Terms])) OR ("non-intubated"[Other Term])) OR ("non-intubated"[Title/Abstract]))) AND (((((("hypoxic respiratory failure"[MeSH Terms]) OR ("hypoxic respiratory failure"[MeSH Terms])) OR ("hypoxic respiratory failure"[Title/Abstract])) OR ("hypoxemic respiratory failure"[MeSH Terms])) OR ("hypoxemic respiratory failure"[Other Term])) OR ("hypoxemic respiratory failure"[Title/Abstract])) OR ("hypoxemia"[MeSH Terms])) OR ("hypoxemia"[Other Term])) OR ("hypoxemia"[Title/Abstract])) OR ("Acute Respiratory Distress Syndrome"[MeSH Terms])) OR ("Acute Respiratory Distress Syndrome"[Other Term])) OR ("Acute Respiratory Distress Syndrome"[Title/Abstract])) OR ("Acute Respiratory Distress Syndrome (ARDS)"[MeSH Terms])) OR ("Acute Respiratory Distress Syndrome (ARDS)"[Other Term])) OR ("Acute Respiratory Distress Syndrome (ARDS)"[Title/Abstract])) OR ("ARDS"[MeSH Terms])) OR ("ARDS"[Other Term])) OR ("ARDS"[Title/Abstract])) OR ("acute hypoxemic respiratory failure"[MeSH Terms])) OR ("acute hypoxemic respiratory failure"[Other Term])) OR ("acute hypoxemic respiratory failure"[Title/Abstract])) OR ("acute hypoxic respiratory failure"[MeSH Terms])) OR ("acute hypoxic respiratory failure"[Other Term])) OR ("acute hypoxic respiratory failure"[Title/Abstract])) OR ("respiratory distress syndrome"[MeSH Terms])) OR ("respiratory distress syndrome"[Other Term])) OR ("respiratory distress syndrome"[Title/Abstract])) OR ("Respiratory Insufficiency"[MeSH Terms])) OR ("Respiratory Insufficiency"[Other Term])) OR ("Respiratory Insufficiency"[Title/Abstract])) OR ("Respiratory Distress Syndromes"[MeSH Terms])) OR ("Respiratory Distress Syndromes"[Other Term])) OR ("Respiratory Distress Syndromes"[Title/Abstract])) OR ("Human ARDS"[MeSH Terms])) OR ("Human ARDS"[Other Term])) OR ("Human ARDS"[Title/Abstract])) OR ("Adult Respiratory Distress Syndrome"[MeSH Terms])) OR ("Adult Respiratory Distress Syndrome"[Other Term])) OR ("Adult Respiratory Distress Syndrome"[Title/Abstract])) | 522 |
| Scopus | (TITLE-ABS-KEY("hypoxic respiratory failure") OR TITLE-ABS-KEY("hypoxemic respiratory failure") OR TITLE-ABS-KEY("hypoxemia") OR TITLE-ABS-KEY("Acute Respiratory Distress Syndrome") OR TITLE-ABS-KEY("Acute Respiratory Distress Syndrome (ARDS)") OR TITLE-ABS-KEY("ARDS") OR TITLE-ABS-KEY("acute hypoxemic respiratory failure") OR TITLE-ABS-KEY("acute hypoxic respiratory failure") OR TITLE-ABS-KEY("respiratory distress syndrome") OR TITLE-ABS-KEY("Respiratory Insufficiency") OR TITLE-ABS-KEY("Respiratory Distress Syndromes") OR TITLE-ABS-KEY("Human ARDS") OR TITLE-ABS-KEY("Adult Respiratory Distress Syndrome")) AND (TITLE-ABS-KEY("COVID-19 pandemic") OR TITLE-ABS-KEY("COVID19 pandemic") OR TITLE-ABS-KEY("COVID-19 pandemics") OR TITLE-ABS-KEY("Severe Acute Respiratory Syndrome Coronavirus 2 infection") OR TITLE-ABS-KEY("SARS Coronavirus 2 Infection") OR TITLE-ABS-KEY("COVID-19 Virus Disease") OR TITLE-ABS-KEY("COVID19 Virus Disease") TITLE-ABS-KEY("COVID-19 Virus Diseases") OR TITLE-ABS-KEY("2019-nCoV Infection") OR TITLE-ABS-KEY("2019-nCoV Infections") OR TITLE-ABS-KEY("COVID19") OR TITLE-ABS-KEY("Coronavirus Disease 2019") OR TITLE-ABS-KEY("Coronavirus Disease-19") OR TITLE-ABS-KEY("Coronavirus Disease 19") OR TITLE-ABS-KEY("SARS-CoV-2 Infection") OR TITLE-ABS-KEY("SARS-CoV-2 Infections") OR TITLE-ABS-KEY("SARS CoV 2 Infection") OR TITLE-ABS-KEY("2019 Novel Coronavirus Disease") OR TITLE-ABS-KEY("2019 Novel Coronavirus Infection") OR TITLE-ABS-KEY("2019-nCoV Disease") OR TITLE-ABS-KEY("2019-nCoV Diseases") OR TITLE-ABS-KEY("2019 nCoV Disease") OR TITLE-ABS-KEY("COVID-19 Virus Infection") OR TITLE-ABS-KEY("COVID-19 Virus Infections") OR TITLE-ABS-KEY("SARS Coronavirus 2") OR TITLE-ABS-KEY("Coronavirus Disease 2019 Virus") OR TITLE-ABS-KEY("2019 Novel Coronavirus") OR TITLE-ABS-KEY("2019 Novel Coronaviruses") OR TITLE-ABS-KEY("Severe Acute Respiratory Syndrome Coronavirus 2") OR TITLE-ABS-KEY("Wuhan Seafood Market Pneumonia Virus") OR TITLE-ABS-KEY("SARS- CoV-2 Virus") OR TITLE-ABS-KEY("SARS- CoV-2 Viruses") OR Terms and conditions Privacy policy Copyright © 2022 Elsevier B.V. All rights reserved. Scopus® is a registered trademark of Elsevier B.V. TITLE-ABS-KEY("SARS CoV 2 Virus") OR TITLE-ABS-KEY("2019-nCoV") OR TITLE-ABS-KEY("COVID-19 Virus") OR TITLE-ABS-KEY("COVID 19 Virus") OR TITLE-ABS-KEY("COVID-19 Viruses") OR TITLE-ABS-KEY("Wuhan Coronavirus") OR TITLE-ABS-KEY("COVID 19 Virus") OR TITLE-ABS-KEY("COVID 19 Viruses")) AND (TITLE-ABS-KEY("awake proning") OR TITLE-ABS-KEY("prone") OR TITLE-ABS-KEY("prone position") OR TITLE-ABS-KEY("proning") OR TITLE-ABS-KEY("prone positioning") OR TITLE-ABS-KEY("prone oxygenation") OR TITLE-ABS-KEY("awake prone position") OR TITLE-ABS-KEY("self proning") OR TITLE-ABS-KEY("self-proning") OR TITLE-ABS-KEY("early awake prone") OR TITLE-ABS-KEY("awake prone positioning") OR TITLE-ABS-KEY("non-intubated")) | 101 |
| Web of Science | ("hypoxic respiratory failure" OR "hypoxemic respiratory failure" OR "hypoxemia" OR "Acute Respiratory Distress Syndrome" OR "Acute Respiratory Distress Syndrome (ARDS)" OR "ARDS" OR "acute hypoxemic respiratory failure" OR "acute hypoxic respiratory failure" OR "respiratory distress syndrome" OR "Respiratory Insufficiency" OR "Respiratory Distress Syndromes" OR "Human ARDS" OR "Adult Respiratory Distress Syndrome") AND ("COVID-19 pandemic" OR "COVID19 pandemic" OR "COVID-19 pandemics" OR "Severe Acute Respiratory Syndrome Coronavirus 2 infection" OR "SARS Coronavirus 2 Infection" OR "COVID-19 Virus Disease" OR “COVID19 Virus Disease" OR "COVID-19 Virus Diseases" OR  "2019-nCoV Infection" OR "2019-nCoV Infections"  OR  "COVID19" OR  "Coronavirus Disease 2019" OR  "Coronavirus Disease-19" OR "Coronavirus Disease 19" OR "SARS-CoV-2 Infection" OR "SARS-CoV-2 Infections" OR  "SARS CoV 2 Infection"  OR  "2019 Novel Coronavirus Disease" OR  "2019 Novel Coronavirus Infection" OR "2019-nCoV Disease" OR "2019-nCoV Diseases" OR "2019 nCoV Disease" OR  "COVID-19 Virus Infection" OR  "COVID-19 Virus Infections" OR "SARS Coronavirus 2" OR "Coronavirus Disease 2019 Virus" OR "2019 Novel Coronavirus" OR  "2019 Novel Coronaviruses" OR  "Severe Acute Respiratory Syndrome Coronavirus 2" OR "Wuhan Seafood Market Pneumonia Virus" OR  "SARS- CoV-2 Virus" OR  "SARS- CoV-2 Viruses" OR  "SARS CoV 2 Virus" OR  "2019-nCoV" OR "COVID-19 Virus" OR  "COVID 19 Virus"  OR "COVID-19 Viruses" OR "Wuhan Coronavirus" OR "COVID 19 Virus" OR "COVID 19 Viruses") AND ( "awake proning" OR  "prone" OR  "prone position" OR  "proning" OR "prone positioning" OR "prone oxygenation" OR "awake prone position" OR "self proning" OR "self-proning" OR "early awake prone" OR "awake prone positioning" OR "non-intubated") | 247 |
