# Supplementary material for: Prone positioning effect on tracheal intubation rate, mortality and oxygenation parameters in awake non-intubated severe COVID-19-induced respiratory failure: a review of reviews
Source: Eur J Med Res. 2024 Jan 20;29:63. doi: 10.1186/s40001-024-01661-6 (PMC10799467; doi:10.1186/s40001-024-01661-6)
Supplement: Supplementary file 2 — Additional file 2: Table S2. Results and conclusions of the included systematic reviews and meta-analyses. [file 40001_2024_1661_MOESM2_ESM.docx]

**Table S2.** Results and conclusions of the included systematic reviews and meta-analyses.

| Author/year/ reference | Results | Conclusions |
| --- | --- | --- |
|  |  |  |
| Fazzini et al., 2021 [18] | significant improvement in the PaO2/FiO2 ratio after PP, lower mortality in the group placed in the PP, unchanged tracheal intubation rate and a median of 4 h overall PP tolerance time | *APP can improve oxygenation amongst non-intubated patients with AHRF when applied for at least 4 h over repeated daily episodes. *APP appears safe, but the effect on TI and survival remains uncertain. |
| E.X. Chua et al., 2021 [19] | In comparison to SP, PP significantly improved the PaO₂/FiO₂ and SpO₂, which were associated with lower mortality, with no difference in TI rate | PP improved the PaO₂/FiO₂ ratio with better SpO₂ than SP in COVID-19 patients. |
| Schmid et al., 2022 [20] | *APP compared to SP probably decreases TI rate but may have little or no effect on mortality | APP seems to be an advantageous supportive measure for non-intubated patients with respiratory failure due to COVID-19. |
| Pavlov et al., 2022 [21] | APP did not result in lower intubation or mortality rates, despite significant reported improvements in oxygenation parameters. | High selectivity of patients, inconsistency of PP in published reports and heterogeneity of outcomes emphasize the need for RCT. APP cannot be presently recommended as a standard of care. |
| Ponnapa Reddy et al., 2021 [22] | Significant improvements in Pao2/Fio2 ratio, PaO2, and O2sat, decrease in RR, and no difference in TI post PP in and out of the ICU. No major adverse event was recorded in small studies that reported them. | There was a variable but significant improvement in oxygenation variables with PP (feasible and safe by trained staff) in  non-intubated adult patients with COVID-19–related hypoxemia. |
| Sryma PB et al., 2021 [23] | There was a significant improvement in PaO2/FiO2 ratio, PaO2, and SpO2 during APP. | APP in non-intubated patients with COVID-19 AHRF might be associated with a reduction in the need for TI and improvement in oxygenation. However, its effect on reducing mortality is still unclear. |
| M. T. Awad et al., 2021 [24] | There was no significant difference between APPS compared to non-PP awake patients regarding intubation rate. | Early APP does not significantly impact non-intubated COVID-19 patients with respiratory failure. |
| Beran et al., 2021 [25] | There was a significant reduction in the mortality  the rate in the APP group compared to control with no significant effect on intubation or length of hospital stay. | APP can potentially reduce the in-hospital mortality rate in COVID-19 subjects with hypoxia without a significant effect on the need for intubation or length of hospital stay. |
| Cardona et al., 2021 [26] | The intubation rate was 28%, and the mortality rate was 14% among hypoxic patients with COVID-19 who underwent APP. | APP is a practical and promising intervention for patients requiring supplemental O2 or NIV due to COVID-19 and may serve to prevent TI. |
| R. S. Cruz et al., 2021 [27] | Significant differences in TI in favor of APP but no differences in mortality. Regarding TI, based on sensitivity analysis and excluding the study with the greatest weight, we found that benefit is not maintained. | Because of the low quality of evidence, the current data do not allow us to draw conclusions regarding the benefit of oxygenation, although most studies show an improvement in oxygenation with APP. |
| Jie Li et al., 2022 [28] | Significantly reduced need for TI with APP compared with SP in overall population and among patients who received advanced respiratory support(i.e., HFNO or NIV) in ICU but not in non-ICU settings. | The APP should be used in patients who have AHRF due to COVID-19, require advanced respiratory support, or are treated in the ICU. |
| W Tan et al., 2021 [29] | The aggregated TI and mortality rates were 33% and 4%, respectively, and the intolerance rate was 7%. PP significantly increased PaO2/FiO2 and SpO2, whereas it reduced RR. | The PP could improve oxygenation both in COVID-19 and non-COVID-19 patients and reduce the RR of awake patients with non-intubation AHRF or ARDS. |
| G. T. Chilkoti  et al., 2021 [30] | Awake PP with HFNC was found to be a promising technique in COVID19 | We encourage the use of early APP in the management of COVID-19 disease |
| S. Anand et al., 2020 [31] | The TI and mortality rates were 23.80% and respectively. APP improved oxygenation parameters (SpO_2_, P/F ratio, PO^2^ and SaO_2_). | We recommend early and frequent APP in COVID-19-associated ARDS; however, RCTs are needed before any definite conclusions are drawn. |
| Parashar et al., 2021 [32] | TI rate and mortality rates were 27.1% and 13.3%, respectively. | APP is probably safe and effective in enhancing oxygenation in non-intubated COVID-19 patients. |
| Alhazzani et al., 2021 [33] | All reports showed an improvement in oxygenation while in PP;  however, the magnitude of improvement was imprecise. | There is insufficient evidence to issue a recommendation on using APP in non-intubated adults with severe COVID-19. |
| Ko et al., 2021 [34] | PP improves oxygenation, reduces mortality and decreases the requirement of IMV in patients with ARDS. | Given the benefits, low cost and easy implementation, PP could be implanted in patients with AHRF due to COVID-19 (GRADE IIC). |
| Senderovich H et al., 2022 [35] | Decreased mean RR from 28.4 ±3.5 breaths/min to 21.3 ± 1.3 breaths/min and an improvement in oxygen saturation during PP compared with baseline. | The true efficacy of the prone position is unknown. Additional supportive strategies and PP should be considered if the benefits of treatment outweigh the adverse effect on older patients. |

AMSTAR: A Measurement Tool to Assess systematic Reviews, PaO2/FiO2: Pressure of Arterial Oxygen to Fractional Inspired Oxygen Concentration, PP: Prone Position, SP: Supine Position, APP: Awake Prone Position, SpO₂: Spot Oxygen Saturation, RR: Respiratory Rate, HFNO: High Flow Nasal Oxygen, NIV: Non-Invasive Ventilation, ICU: Intensive Care Unit, ARDS: Acute Respiratory Distress Syndrome, AHRF: Acute Hypoxic Respiratory Hypoxemic Respiratory Failure.
